# The impact of the COVID‐19 pandemic on the mental health and well‐being of children and young people

**DOI:** 10.1111/chso.12430

**Published:** 2020-11-28

**Authors:** Helen Cowie, Carrie‐Anne Myers

**Affiliations:** ^1^ Faculty of Health and Medical Sciences University of Surrey Surrey UK; ^2^ Department of Sociology, City University of London London UK

**Keywords:** children and young people, connectedness, COVID‐19, emotional well‐being, mental health

## Abstract

The COVID‐19 pandemic has had an enormous impact across the world. In this discussion paper, we examine the effect that lockdown has had on the mental health and well‐being of children and young people. We write from a UK perspective in the light of the international evidence. Many of the discussion points raised resonate globally. We discuss how these issues can be dealt with and set out potential solutions as we emerge from this global crisis.

## INTRODUCTION

The COVID‐19 pandemic, the biggest health crisis for generations, has affected more than 200 countries around the world. In China, for example, whole cities were placed under mass quarantine, with many individuals returning from other countries being required to self‐isolate at home. There were precedents for such mass restrictions, for example, during the 2003 severe acute respiratory syndrome (SARS) outbreak and the 2014 Ebola outbreak in West African countries. Accordingly, in March 2020, the government of the United Kingdom (UK) (comprising England, Northern Ireland, Scotland and Wales) took drastic steps to contain the virus, including restriction on movement, lockdown of people in their homes (apart from one brief outing per day for exercise and necessary trips to buy food and for medical purposes) unless they were deemed to be key workers, and the closure of most workplaces, schools and shops, other than those selling essential supplies.

During lockdown, most people were confined to home from early March 2020. At the time of writing, in autumn 2020, restrictions that were eased over the summer have been tightened again with the introduction of new lockdowns, which vary from region to region within the UK. For those children and young people who did not have access to a garden or a park, or who lived in cramped conditions, this period was particularly trying. Typically, youngsters in this situation spent all day with their family or others who live in their household. They could only “see” their peers through online platforms or by smartphone. Some, but not all, schools sent out regular homework so parents/caregivers became teachers, whether they had any aptitude for this role or not, or access to internet resources necessary to carry out educational tasks.

These extreme measures were justified on the grounds that they would save lives and prevent unsustainable pressure on the National Health Service (NHS). Despite this action, the death toll at the time of writing reached over 60,000.

The pandemic came at a time when, throughout the world, many educators and parents/caregivers were already concerned about the rising levels of stress being experienced by children and young people as they encounter pressure to succeed academically (Aveyard, 2018; Humphrey, 2018), as well as high expectations from the peer group, fuelled by the social media, around physical appearance and popularity, linked to fear of embarrassment and shaming (McLoughlin et al., 2018). Additionally, there was growing evidence of the negative health consequences arising from acts of bullying and cyberbullying, including increased levels of anxiety, depressive symptoms, poor self‐worth, social isolation and loneliness, psychosomatic complaints, suicidal ideation and suicide attempts (Al‐Ghabban, 2018; Cowie & Myers, 2018).

The question which we pose is “What impact has lockdown during the current COVID‐19 pandemic had on the mental health and emotional well‐being of children and young people in the UK, bearing in mind that this comprises England, Northern Ireland, Scotland and Wales?” To answer this question, we explored the following:

- international research findings derived from investigations into previous epidemics;
- preliminary findings from on‐going research into the impact on mental health of lockdown and quarantine;
- reports from charities, such as YoungMinds, Barnardo's and YouthLink Scotland, and spokespersons for youth, such as the UK Children's Commissioner,
- reflections on the crisis in the media;
- self‐help guides for parents/caregivers during the crisis.

These sources revealed the multiple layers of challenges that the pandemic caused to the mental health and well‐being of children and young people. Some were already there, embedded in society as a cause for concern, while others emerged as a direct consequence of the lockdown. We discuss the extent to which views are based on sound evidence and consider the role of the family and society as we emerge from this global crisis.

## EVIDENCE FROM INVESTIGATIONS INTO THE IMPACT OF QUARANTINE/SOCIAL DISTANCING ON MENTAL HEALTH DURING EPIDEMICS

International research evidence indicates that pandemics have an extremely negative impact on mental health, with children and young people being especially at risk due to their limited understanding of the event. This has led to an exceptional increase in anxiety and other mental health problems among children and young people with an accompanying decrease in emotional well‐being.

As indicated by the results of international research, closure of schools and limited access to friendship groups can cause acute anxiety and stress in young people (Jiao et al., 2020; Lee, 2020). Furthermore, frequent exposure to media coverage of the crisis can make the mental distress even worse (Centre for Disease Control, 2020; Dalton et al., 2020). Brooks et al. (2020) carried out a rapid review of the possible psychological costs for individuals during an epidemic of quarantine, which they defined as separation and restriction of movement of people who have potentially been exposed to a contagious disease. The studies that met their inclusion criteria were carried out across 10 countries, with reference to outbreaks of SARS, Ebola, the H1N1 influenza pandemic, Middle East Respiratory Syndrome (MERS) and equine influenza. The results indicated that the longer the quarantine, the poorer the mental health, in particular, the greater the reporting of post‐traumatic stress syndrome (PTSD). Overall, the psychological impact was substantial and was likely to continue for a significant length of time after quarantine ended. The researchers emphasised the need for policy‐makers and politicians to communicate frequently and meaningfully with the people whose liberty had been constrained in this way.

While the participants in the Brooks et al. (2020) study were mainly adults, the review by Imran et al. (2020) focussed specifically on the impact of COVID‐19 social distancing on the mental health of children and young people in Pakistan. They noted an increase in feelings of anxiety and uncertainty, as well as heightened risk of bullying due to excessive use of electronic and social media. Furthermore, they noted, social distancing in an abusive home resulted in increased risk of child abuse, neglect and exploitation. They recommended that parents/caregivers safeguard their children's mental health by reassuring them in age‐appropriate ways, educating them about the situation, and maintaining daily routines. They concluded that exposure to panic‐inducing news on the media should be avoided and a positive use of social media should be encouraged, for example, by forming online support groups.

In England, the ongoing Co‐SPACE Study (2020) is tracking the mental health of children and young people aged 4–16 years throughout the COVID‐19 crisis by means of a survey that is completed on a monthly basis by parents/carers and young people (if aged 11–16 years). (At the time of writing, over 11,500 parents/carers have taken part in the survey.) The first report of findings at the beginning of lockdown indicated that parents/carers experienced stress around combining work with their children's well‐being as well as worry about family/friends outside the household. These early findings pointed to an increase in mental health difficulties among younger children during lockdown, expressed through emotional, behavioural and restlessness/attentional difficulties. The situation for adolescents, however, was different. While parents/carers reported an increase in restlessness/attentional difficulties, the young people themselves reported no change in their own emotional or behavioural and restlessness/attentional difficulties. Parents/carers of children with SEN and those with a pre‐existing mental health difficulty reported a reduction in their child's emotional difficulties and no change in behavioural or restlessness/attentional difficulties. The latest report (at time of writing) (Co‐SPACE, 2020) highlights the finding that emotional and restless/attentional difficulties (and behaviour difficulties for primary school children) were consistently elevated over a month of lockdown among children and young people from low income households compared to those from higher income households. Children and young people from lower income families had higher levels of emotional difficulties, such as feeling unhappy or worried, being clingy and experiencing physical symptoms associated with anxiety than those from high income households. In other words, these findings indicate that the pandemic has already had a significant impact on the mental health of children and young people, with particular effect on the most deprived families and their children.

In August^,^ 2020, 275 former world leaders, economists and educationalists called on the G20 nations and other countries for urgent action to avoid a ‘COVID generation’ affecting tens of millions of children and young people with no hope of an education, recommending priority to 30 million children and young people who, according to a UNESCO (2020) report, may never return to school (Relief Web International, 2020). They also warned that the world's poorest children and young people have been locked out of learning and denied internet access, and that with the loss of free school meals hunger is growing. Orbach (2020) had already expressed concerns over school closures as they began to happen during the COVID outbreak, stating that they carry high social and economic costs for people across communities. The impact, however, is particularly severe for the most vulnerable and marginalised boys and girls and their families in all four regions of the UK. The resulting disruptions exacerbate already existing disparities within the education system but also in other aspects of their lives.

## REPORTS FROM CHARITIES AND OTHER NGOS AND SPOKESPERSONS FOR YOUTH

The office of the Children's Commissioner (2020a), using data from the 2017/18 cohort of children and young people in the National Pupil Database, quantifies the numbers receiving high levels of support for their needs as well as those where their support is unclear or they have lost contact with mainstream services, or those viewed as ‘falling through the gaps’. They found that in 2017/18 nearly 480,000 teens aged 13–17 met at least one of the following criteria:

- Any identified special educational need or disability (SEND);
- Any Child in Need (CIN) referral/episode during the year;
- Any fixed exclusions during the year;
- Any permanent exclusions during the year;
- High levels of absence during the year (missing at least 15% of school);
- Any time at a pupil referral unit (PRU) during the year;
- Dropping out of the school system in Year 11;
- Missed at least an entire term of school in the last 2 years.

Of this group, 140,000 teens had two or more risks, 46,000 had three or more risks, and 14,500 had four or more risks—signals of children and young people at higher risk of future educational failure and unemployment, as well as of falling into crime and criminal exploitation and having long‐term consequences on their mental health and well‐being (Farrington et al., 2016).

COVID‐19 has increased many of the risks facing children and young people, not just in terms of the epidemiological risk, but also in terms of the additional risks that the lockdown itself has created, such as an increased risk of poor mental health, exposure to domestic violence and addiction in the home, and exposure to exploitation. These risks have been exacerbated by the closures of schools and access to other services.

Although the effects of the pandemic will have been particularly acute on the teenagers who were already vulnerable before COVID‐19, especially those who were ‘falling through the gaps’, with schools closed to most pupils for half an academic year, and face‐to‐face social care provision being stopped, these young people risk becoming even more ‘invisible’ than before. There will also be a new wave of potentially struggling students, who were not previously experiencing mental health or well‐being problems, but who will, as a consequence of lockdown, run the risk of becoming a ‘lost generation’.

For example, in a survey of 2,421 young people in Scotland carried out by the Scottish Youth Parliament, YouthLink Scotland and Young Scot (2020), nearly two‐fifths of the respondents said that they were moderately or extremely concerned about their own ‘mental well‐being’. Similarly, Girlguiding (2020), which has been tracking girls’ and young women's views through the Girls’ Attitudes Survey for over a decade, surveyed 6,678 girls and young women aged between 4 and 18 years across the UK to find out how the pandemic crisis and lockdown was affecting them. They found that 33% of girls between 4 and 10 years reported that they felt sad most of the time while 34% reported that they felt lonely most of the time. 45% of girls aged from 11 to 14 reported feeling stressed and 42% reported feeling worried most of the time. Young women between 15 and 18 particularly reported feeling worried, stressed and overwhelmed, with concerns about their future, their education, the cancellation of their exams and the perception that social isolation was putting a strain on their relationships.

Other sources presented a similarly dark picture, with an emphasis on conflict, anger, frustration and fear (UNESCO, 2020). For example, Barnardo’s (2020) co‐produced research in England with a group of young people which reported a significant impact on mental health. In another study in England, Simpson (2020) found that Asian parents/caregivers reported experiencing hostility and racism from their local community based on misperceptions of the causes of the pandemic, including accounts by children of Chinese heritage of experiencing racist bullying by peers who used cruel terms such as ‘coronavirus spreader’ or ‘contagious’. Police in all regions of the UK reported a disturbing rise in incidents of domestic violence. Family doctors reported a silent epidemic of despair among their patients, indicated by panic attacks, sleeplessness, suicidal thoughts, alcohol‐induced injuries and assaults. Similarly, helplines such as ChildLine and Samaritans, reported an increased volume of calls. Some parents/caregivers, experiencing acute anxiety about loss of income and heightened risk of unemployment, found it hard to focus on the well‐being of their children. For the parents/caregivers of children with special educational needs, lockdown seemed to be extremely challenging and the reduction in professional support from schools and healthcare services was keenly felt.

Furthermore, across the UK, in two surveys carried out by YoungMinds (2020a, 2020b) with adolescents who were already experiencing mental health difficulties, the participants reiterated their need for structure in their days, access to the creative arts and regular communication with friends and other social groups. However, the YoungMinds research also indicated the huge difficulties that some families would experience in implementing such recommendations. In the first, YoungMinds (2020a) surveyed 2,111 adolescents with a history of mental health needs between 20 March and 25 March^,^ 2020 (when restrictions were tightened even more). Seventy‐four per cent reported that they had benefited greatly from continuing access to professional help, whether from NHS mental health services, private practitioners, school and university counsellors or charities. The main sources of help that were experienced as beneficial included:

- Social connection with friends;
- Taking exercise;
- Playing or listening to music;
- Being outdoors in nature;
- Spending time with pets;
- Writing/ keeping a journal.

The activity that increased anxiety the most was reading or watching the news which was dominated by updates on COVID‐19.

The young respondents overwhelmingly supported the Government's decision to close schools and universities, to recommend social distancing and ban public gatherings. At the same time, the respondents reported increased anxiety, problems with sleeping, panic attacks and, among those who already self‐harmed, increased urges to do so. Respondents reported that the public health measure had made their mental health ‘a little bit worse’ (51%) or ‘much worse’ (32%). The survey revealed four major sources of anxiety.

Concerns for the health of their families, fear of transmitting the virus and worry for the health of family members who were key workers in places where there was a heavy risk of spreading or catching the disease.

Closure of schools which, to many of the young respondents, offered predictable routines and a safe environment, as well as a source of emotional support from peers, teachers, counsellors and healthcare professionals. For some, school or university was viewed as a haven from violent or difficult households; for them the closure was extremely difficult.

Uncertainty about grades since key examinations were cancelled or postponed. Home learning was experienced as stressful by many of the respondents because of such factors as lack of online resources in the home, closure of libraries, lack of peace and space for study and difficulty in concentrating without the framework usually provided by school.

Loss of routine and activities, such as exercise or dance classes, that usually provided them with a coping mechanism for dealing with their mental health issues. Long stretches of time spent at home led many of the young respondents to brood on their own problems, think negatively about themselves, and dwell on harmful strategies for dealing with distress.

A recurring theme in this survey was the lack of social connectedness with friends and trusted adults outside the home. This was especially salient for young people without access to the internet. Even those who could communicate with their friends online reported a lack of intimacy and closeness in these exchanges, so heightening their sense of loneliness and isolation.

The second YoungMinds survey (YoungMinds, 2020b), conducted with 1,850 parents/caregivers, elicited worries about the long‐term impact of COVID‐19 on their children's mental health, and described the challenges they faced in finding support. The key findings were:

67% expressed concern about the long‐term impact of the coronavirus on their child's mental health. This rose to 77% among those whose children had required mental health support in the previous three months of the study, just as the lockdown was announced.

29% disagreed that there was enough support (information, helplines and resources) available to help them and their child get through this time, and 24% said that they would not know where to turn for advice.

Among respondents whose children had received mental health support in the previous three months, 25% said that their child was no longer able to access it, but still needed it.

66% reported a negative impact on their own mental health.

These surveys provide evidence about issues within the home that some children and young people were expected to navigate during the pandemic.

## THE IMPACT OF DOMESTIC VIOLENCE ON THE MENTAL HEALTH OF CHILDREN AND YOUNG PEOPLE

Further risks to young people's safety and well‐being emerged from reports about domestic violence, with disturbing evidence that for some young people the family household was a dangerous space to occupy. A global surge in domestic abuse was reported during the coronavirus pandemic, as those living with domestic violence were put at increased risk by lockdown rules (Humphreys et al., 2020; Imran et al., 2020).

Similarly, in the UK, the Home Office (2020) reported that calls to the national domestic abuse helpline run by the charity Refuge were substantially higher after lockdown than before; analysis of online traffic by Chayn, a website that addresses gender‐based violence, showed that visitors to its website had more than trebled during lockdown in comparison with the same period last year.

Statistics like these indicated serious risks for the mental health and well‐being of the young people who live within these households. Children are often the silent witnesses of such violence and the extent of the impact on them was emerging as a key cause for concern. Reports of children being abused at home surged by almost a third after lockdown was imposed. The National Society for the Prevention of Cruelty to Children (NSPCC) (2020) fielded an average of one call an hour, with over 1,500 calls being taken since the restrictions were introduced. This equates to an increase of 32% from before lockdown. In some cases, fears about the virus were exploited by the perpetrators to withhold access to children, cut off contact to family and friends, and monitor movement under the pretext of keeping them safe from the virus. Those affected said this made it difficult to leave home and speak out.

## GUIDING PARENTS/CAREGIVERS DURING THE PANDEMIC—THE IMMEDIATE ROLE OF THE FAMILY

Reports in the media in all four countries of the UK presented both positive and negative aspects of lockdown (e.g. Francis, 2020). The internet abounded with websites offering online yoga, keep‐fit classes, art class, online choirs, dance workshops, and many other opportunities for people to express their creativity during the enforced restrictions on their lives through lockdown, with an explosion of videos and online platforms with guidance on arts, crafts, gardening, storytelling, observing nature and music‐making. Some parents/caregivers in this situation reported that they recognised the benefits of having time to enjoy their relationships and discover new, co‐operative activities to engage in with their children that they had not previously found time or space to do.

As it became clear that the home would be the main setting in which formal education was to take place, some academics and practitioners produced guides and information. Health agencies, such as the WHO and UNICEF, and governments produced guidelines and information sheets for parents/caregivers about the potential risks to the mental health of children and young people. Charities, such as *Place2be* and *YoungMinds*, quickly produced online guidance for children and young people experiencing mental health difficulties at this challenging time.

Similarly, Long and Evans (2020) devised a lockdown guide for all parents/caregivers, with additional resources for children and young people with special needs. The authors acknowledged the challenge facing parents/caregivers, whether they were at home all day with their children or out at work. Long and Evans (2020) emphasised the necessity for parents/caregivers to ensure for their children that two fundamental needs were met: the *need to feel safe* and the *need to belong*. They highlighted the fact that the constant presence of distressing news about the COVID‐19 pandemic on the media has the potential to threaten the need to feel safe. While they acknowledged that children could not be shielded from all the negative information that came out daily, they emphasised the need for parents/caregivers to provide reassurance and comfort as well as honest, age‐appropriate information about the dangers posed by the pandemic, and predicted that, without such support, many children and young people would experience such difficulties as uncertainty about the disease, the fear of becoming ill or seeing a family member suffer, the loss of the rhythms of everyday routines, the difficulty involved in connecting with other people that are usually close to them, and the absence of schooling. Each one of these factors, they argued, can pose a risk to the mental health of children and young people and their families. These recommendations were closely in line with the guidelines for parents/caregivers offered by Imran et al. (2020) based on their review of the impact of quarantine on children and young people, and the crucial role of the family. To facilitate children's sense of safety and connectedness, Long and Evans (2020) recommended:

*Setting routines*: to help structure the day.

*Making memories*: creating opportunities to discuss the good news and the positive things that the family did during lockdown.

*Having fun*: doing simple things, such as cooking together, playing boardgames together, doing crafts and drawing.

*Using social media*: playing online games with friends; chatting with friends; arranging online parties for special occasions.

*Dealing with conflict within the family*: parents/caregivers need to remain calm and to develop mediating strategies to defuse arguments and disagreements between family members.

*Managing anxiety*: understandably, young people are anxious during the COVID‐19 pandemic, so parents/caregivers have a key role in listening with understanding to their children's fears about the disease and reassuring them by taking their worries seriously and answering their questions with understanding and empathy.

*Building self‐esteem*: it is important for parents/caregivers at a difficult time to take time to build up the child's sense of worth. It is also useful for parents/caregivers to be consistent in their use of positive thinking about the child's talents and abilities.

Further affirmation of the need for structure and stimulation in children's lives also came from preliminary UK research reports. Girlguiding (2020), for example, in their survey of nearly 7,000 girls and young women identified some positive aspects of lockdown, such as awareness by the young people of improvements to the environment, as well as appreciation of the time spent with their families doing activities together that they enjoyed and finding the time to take up new hobbies. Most girls in this survey took part in community action, such as the weekly applause for NHS workers (96%), putting rainbows or posters in their windows (90% of 4–10‐year‐olds), and fundraising for charity (30% of 15–18‐year‐olds).

## IS THERE A WAY FORWARD?

The pandemic continues with restrictions having uneven impact on the lives of children and young people. It is too early to draw firm conclusions about the impact of COVID‐19 on the mental health and well‐being of children and young people in advance of publication of the ongoing research that is taking place. Here we suggest some ways forward based on the existing evidence.

### Mental health

Mental health problems are known to have a disproportionately negative impact on the lives of children and their families who are already disadvantaged. Mental health needs to be understood in relation to wider social and structural conditions if we are to develop appropriate responses. Barnardo’s (2020), for example, recommends long‐term change to policy involving the rebalancing of the educational system so that mental health and well‐being are on a par with academic achievement, and a redesign of mental health provision in partnership with the young people themselves towards a continuum of care that includes digital services and alternative therapies. COVID‐19 has highlighted the gulf between pre‐existing health conditions, poverty and the role of the school and children's services in some sections of society (Co‐SPACE, 2020; Fancourt, 2020). In some areas, as highlighted by the Children’s Commissioner (2020a, 2020b), the well‐being of children and young people was not being sufficiently dealt with before the pandemic. This needs to be addressed as a matter of urgency.

### The role of parents/carers

Parents/carers play a crucial part in supporting their children in difficult times such as a pandemic but their capacity to provide emotional, educational and social support must interrelate with school and community. Where the family is living in circumstance of poverty or extreme anxiety about employment, there is greater likelihood of adverse outcomes, since such circumstances make caregiving more difficult and challenging. In conditions of lockdown during a pandemic, some families have poor quality living conditions, lack of good nutrition, lack of space for study, less access to resources like books and the internet, all of which place great barriers on the families as they strive to cope with the challenges facing them during lockdown. The potential for parents/carers to be resilient should not be underestimated but many families need help in surmounting the difficulties that they face.

### Families in the community

In the present COVID crisis, families and communities have great potential to help their children and young people to address the fears and anxieties that arise. As we saw, the pandemic has led to an outpouring of poetry, songs, music, art, theatre, dance that have enabled children and young people throughout the country to find strength within themselves to deal with the crisis. Many young people have made constructive recommendations about interventions that might help during and after lockdown (Barnardo’s, 2020; YoungMinds, 2020b). Self‐help guides like the ones developed by WHO and UNICEF, by educators such as Long and Evans (2020) and researchers such as Imran et al. (2020) might be criticised for offering a model that is unrealistic for many parents/carers. However, we must not forget that the young people themselves highlight physical activity, creative arts and nature as conducive to well‐being, so it is essential to open up pathways for all families so that these valuable resources can be made more widely available to all (Girlguiding, 2020). That is why it is essential for society to provide policies and practices that enable families to create the best and most nurturing environment possible.

### Peer on peer bullying

Although during lockdown the victims of bullying may have been relieved not to have to go to school, as we have shown, some bullying continued, either through social media or face‐to‐face in the community (e.g. Simpson, 2020). The re‐opening of schools offers a new challenge. Schools can develop peer support schemes and other interventions that build on the altruism demonstrated during lockdown and that challenge moral disengagement among those who bully and those who are bystanders (Cowie, 2020). The importance of connectedness in schools as a means for promoting emotional well‐being is widely documented. With the help of supportive networks, children and young people are more likely to develop positive beliefs in themselves and feelings of competence and optimism (McBeath et al., 2018; McLoughlin et al., 2018). Schools play a key role in supporting bullied children and in promoting values that include vulnerable children in the peer group rather than excluding them. For those with existing mental health difficulties, the YoungMinds (2020b) research demonstrates the need for young people to have knowledge about where to go for help, including digital, virtual, text‐based and telephone therapies for young people. The young people themselves also highlight the huge value to their well‐being of the arts, sport and access to nature and open spaces. The YoungMinds (2020b) and Girlguiding (2020) surveys also highlight the positive suggestions from the young people themselves about what would help them most, confirming the critical importance of accessing the voice of the child and listening to the solutions that they propose. The potential for children and young people to demonstrate care and concern for vulnerable peers should not be underestimated and there is huge scope for such schemes as the Diana Foundation Anti‐Bullying Ambassadors to promote the concept of *upstanding* as opposed to *bystanding* in the face of bullying/cyberbullying.

### Risk and protective factors

Risk and protective factors exist at the level of the *family* (e.g. where there is discord and domestic violence, parental physical and mental illness, economic deprivation), the *community* (e.g. where there is poverty, high levels of crime, homelessness, prejudice, discrimination, racial tension), and within the *young person* (e.g. if he/she has an illness, has behavioural difficulties, misuses drugs or alcohol). That is why it is so important to create opportunities for children and young people to develop competencies to deal with the social and emotional difficulties that they are facing during this time of crisis (Cefai et al., 2018; Ungar, 2019). Protective factors include: good relationships within the family, opportunities for the child to develop a sense of mastery, whether in sport, the arts, academic skills and experiences that foster self‐esteem; also access to education, healthcare facilities, wider support networks, community facilities.

## INEQUALITY IN SOCIETY

The pandemic has served to increase the extent of inequalities in our society. Without the school as an institution of support for the vulnerable in society, there will be long‐term problems for children and young people. Furthermore, at the time of writing, levels of homelessness, poverty, lack of access to resources and an increase in the use of foodbanks for many families demonstrate the problems that schools faced when they eventually reopened. Some calls have already been made to the government on how to deal with the aftermath of the pandemic. YoungMinds, based on the research reported above, launched its *Beyond Tomorrow* campaign at the end of May urging local and central governing bodies across the UK to ensure that young people who need mental health advice can get it, that there is increased support for families, and that schools are able to prioritise students’ well‐being. It also calls for continued investment in mental health services as restrictions change, to ensure that young people who have been affected can get ongoing support. Similarly, lockdown has highlighted the need for the law to recognise the extent to which domestic violence affects children and young people. The governing bodies in all four countries of the UK should grasp the landmark opportunity offered by the Domestic Abuse Bill (currently at the Committee stage) and ensure children and young people get the protection and support they need.

## CONCLUSION

There were many issues that children and young people faced before the pandemic and some of these have now been exacerbated by it and remain a priority to be urgently addressed. Unless those responsible for governance, whether local, regional or national in the UK, act now and respond to the issues that have been raised by the charities and practitioners who have seen an increase in their work load and resources in response to the impact of lockdown, there will be thousands of children and young people who are lost within the system (Children’s Commissioner, 2020a, 2020b). Schools play a pivotal role in providing a wealth of support for children and young people, including food, comfort and a refuge from the home and the wider society. The evidence indicates a key role to be played here through creative arts, physical activity, opportunities to act altruistically and access to open spaces.

As Orbach (2020) points out, the pandemic has undoubtedly placed our society in a state of collective trauma. But we have choices. We can suffer it alone as individuals and within our families or we can view it as an opportunity for people to go through it together. As our argument indicates, this is a chance to affirm our deep human need for *connectedness* and to draw on our resources of *strength*, whether within ourselves, or in the people and systems around us to make sure that the UK does not lose a generation, notably our children, our future generation, to COVID‐19 as a consequence of the pandemic.
